# A Real World Study of Aducanumab Treatment in Patients with Early Alzheimer’s Disease in China

**DOI:** 10.1002/alz70861_108194

**Published:** 2025-12-23

**Authors:** Gang Wang

**Affiliations:** ^1^ Department of Neurology, Renji Hospital Affiliated to Shanghai Jiaotong University School of Medicine, Shanghai 200217, Shanghai, Shanghai China

## Abstract

**Background:**

The aggregation of amyloid‐β (Aβ) is widely regarded as a key factor in the pathogenesis of Alzheimer’s disease (AD). The aducanumab was the first anti‐Aβ monoclonal body clinically used in China.

**Methods:**

It was a 12‐month, single‐center, real‐world study to observe the efficacy and safety of aducanumab in early AD in Chinese population. The study involved patients under 80 years of age with mild cognitive impairment (MCI) or mild dementia due to AD with evidence of amyloid on positronemission tomography (PET). Patients received aducanumab treatment via intravenous infusion every 4 weeks (titrated to 10 mg/kg, 12 doses total). The primary outcome was the change from baseline to 12 months on the score of the Clinical Dementia Rating‐Sum of Boxes (CDR‐SB), with higher scores indicating greater decline in function and cognition. Other key secondary outcomes included the change of amyloid burden on PET and brain structure on T1‐weighted imaging and diffusion tensor imaging.

**Results:**

12 participants completed the treatment and clinical assessment. The mean CDR‐SB change from baseline at 12 months was 0.917 (95% confidence interval [CI], ‐0.08 to ‐1.75; *p* =0.034). The cognitive decline predominantly occurred in mild AD patients. The average brain amyloid clearance at 12 months compared to baseline were ‐30.998 centiloids (95% CI, ‐46.74 to ‐15.25; *p* =0.001). Among all the participants, two autosomal dominant AD patients manifested an initial amyloid increase at 6 months followed by subsequent decline at 12 months. Besides, brain structure showed decrease in mean cortical thickness and left hippocampus volume, and increase in choroid plexus and ventricular volume. No amyloid‐related imaging abnormalities (ARIA) was observed during the treatment.

**Conclusion:**

The first anti‐Aβ monoclonal body, aducanumab, was safely used in China. The efficacy of brain Aβ clearance exhibited individual variability, particularly in patients carrying pathogenic gene mutations, where the therapeutic outcomes might be weaker. Such medications may be more effective when recommended for MCI patients, as they can exert their efficacy in earlier stage and help delay cognitive decline.

